# The Second Wave of COVID-19: Clinical Pharmacy Services During a Field Hospital Operation

**DOI:** 10.1177/00185787211032361

**Published:** 2021-07-26

**Authors:** Jessica Mazzone, Krysta Shannon, Richard Rovelli, Racha Kabbani, Angel Amaral, Neil Gilchrist

**Affiliations:** 1 UMass Memorial Medical Center, Worcester, MA, USA; 2 UMass Memorial Health Care, Worcester, MA, USA

**Keywords:** clinical services, dispensing, education, medication safety, medication process

## Abstract

The second wave of COVID-19 emerged in the late fall months in the state of Massachusetts and inadvertently caused a rise in the number of cases requiring hospitalization. With a field hospital previously opened in central Massachusetts during the Spring of 2020, the governor decided to reimplement the field hospital. Although operations were effectively accomplished during the first wave, the reimplementation of the field hospital came with its new set of challenges for operating a satellite pharmacy. Experiences gathered include new pharmacy operation workflows, the clinical role of pharmacy services, introduction of remdesivir treatment, and pharmacy involvement in newly diagnosed diabetes patients requiring insulin teaching. Pharmacy services were successful in adapting to the rapidly growing number in patients with a total of over 600 patients served in a course of 2 months.

## Introduction

In the fall of 2020, publicly reported data indicated that COVID-19 cases were intensifying in the state of Massachusetts. In 1 month, the number of new cases increased from 614 on October 13th, to 1537 on November 11th. During this time, the 7-day average of COVID-19 patients hospitalized increased 2-fold with 661 confirmed patients in the hospitals. It was becoming more apparent that the state needed to develop a strategy to manage the non-ICU hospital bed occupancy on the rise to 73% of hospital capacity across the state.^1^

On November 13th, 2020, the Governor of Massachusetts announced the decision to re-open the field hospital located at a large 50 000 square foot indoor convention center in central Massachusetts in the city of Worcester.^2^ Prior experience from implementing this field hospital in March of 2020 allowed staff to open much more efficiently but required the development of new strategies due to an unexpected expansion in the number of patients admitted.

Within the first 11 weeks of operations of the field hospital the number of COVID 19 surpassed the first wave of 81 933 cases by recording over 321 136 new cases in Massachusetts.^3,4^ During the first surge with 6 weeks of field hospital operations, a total of 161 patients were treated. With just 13 weeks of re-opening, the field hospital treated 642 patients.^2^ As nursing and field hospital staff was doubled in response to the surge of patients, 1 clinical pharmacist and 1 certified pharmacy technician were responsible for maintaining pharmacy services for 60 to 70 patients per day. Despite challenges with the numbers growing exponentially, pharmacy services had an opportunity for innovative changes to clinical and operational workflows.

## Updates to the Field Hospital Pharmacy Operations

The field hospital made some vital changes during its reopening for the second surge of COVID-19. Additional admission criteria were incorporated into patient inclusion factors such as patients with stable chronic cardiovascular conditions, hemodialysis, remdesivir treatment, and COVID-19 Convalescent Plasma treatment. Patients with multiple comorbidities including diabetes mellitus, COPD, asthma, atrial fibrillation, and chronic kidney disease requiring pharmacy intervention and monitoring were admitted to the field hospital in the second re-opening. These new inclusion criteria allowed for the field hospital to serve a greater patient population and open hospital capacity to serve non-COVID-19 patients.

**Figure 1.**
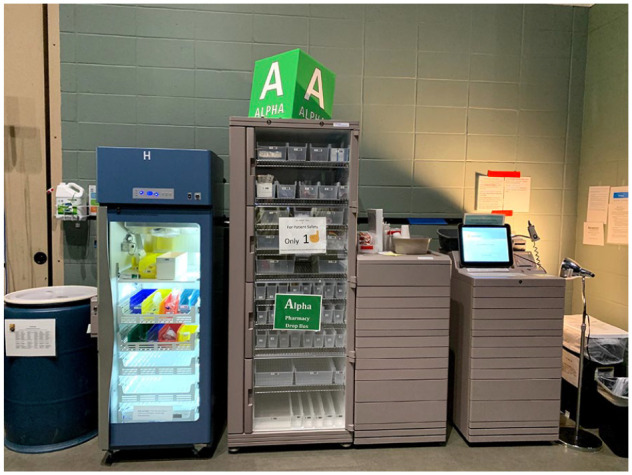
Alpha ADC.

**Figure 2.**
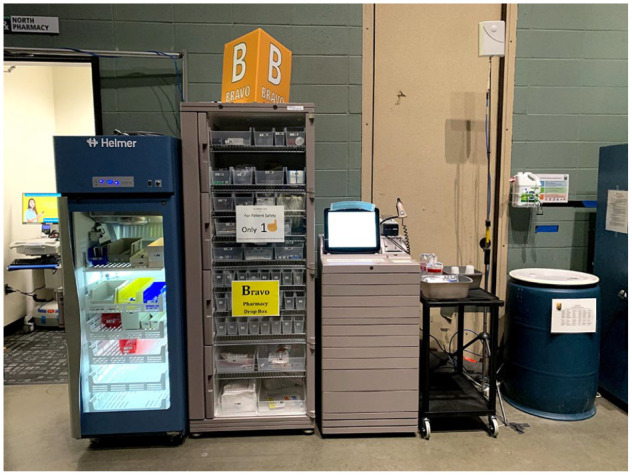
Bravo ADC.

The initial setup pharmacy operation included 2 automated dispensing cabinets (ADCs) and refrigerators with a capacity to serve up to 75 patients. As the daily census of COVID 19 patients increased, 2 additional ADCs and connected refrigerators were added to the operation creating a capacity of up to 150 patients for medication management. The configuration of the ADCs was identical to the previous field hospital operations for nursing to obtain medications from any available ADC. With the unexpected influx in the number of patients, reaching its peak number at a daily census of 87 patients on January 3rd, 2021, the 4 ADC pharmacy operation were not efficient in supporting nursing workflow. To alleviate the issue, the configuration of the ADCs was adjusted to assign each ADC to a specific nursing unit (4 units total in the field hospital). Each ADC was optimized with only the necessary medications for their respective unit and updated daily. The outcome of this measurement resulted in increased efficiency and less time spent searching for medications, thus a greater focus on patient care. Nurse management can attest to these outcomes by noting “shorter lines to the ADCs and nursing no longer pulling medications for multiple patients at a time.” To alleviate the issue, the configuration of the ADCs was adjusted to assign each ADC to a specific nursing unit (Figures 1 & 2; four units total in the field hospital).

## The Call to Action: Clinical Pharmacist Presence in a COVID-19 Field Hospital

The COVID-19 pandemic has required pharmacists to step out of their traditional roles and provide patient-centered care in unique, unconventional settings. This has been portrayed through the opening and operation of hospital-based pharmacy services at COVID-19 field hospitals.^5-7^ Although pharmacy operations at field hospitals have previously been discussed, there is a paucity of data pertaining to objective markers of pharmacist interventions in this setting.

The 1999 report, *To Err is Human*, highlighted the prevalence of preventable medical errors that contributed to as many as 98 000 deaths annually in American hospitals.^8^ Medication-related errors themselves were frequently encountered in hospital systems and accounted for an estimated 2 billion dollars’ worth of increased hospital costs. With training focused on pharmacotherapeutic optimization, pharmacists are in an optimal position to reduce medication-related errors and improve patient outcomes. Over time, the volume of pharmacist interventions and their associated outcomes have been well-documented in the traditional hospital setting. Drug error categories include prescribing, medication administration, monitoring, dispensing, and documentation. Potential drug errors provide opportunities for pharmacist intervention, with rates of recommendation acceptance approaching 90%.^9^ In fact, pharmacist presence on multidisciplinary patient rounds has been shown to significantly reduce patient hospital length of stay and incidence of unplanned readmissions.^10,11^

Central to the training of clinical pharmacists is their positive effect on medication errors and discrepancies. Walker et al conducted a prospective, quasi-experimental study to quantify the effects of pharmacist intervention on health care utilization following discharge. Outcomes including 14-day readmission rates, 30-day readmission rates, and incidence of medication discrepancies in patients receiving pharmacist intervention (n = 358) were compared to controls (n = 366). Incidence of 14-day (12.6% vs 11.5%, *P* = .65) and 30-day readmission (22.1% vs 18%, *P* = .17), assessed using logistic regression, did not differ significantly between groups. Incidence of any medication discrepancy (33.5% vs 59.6%, *P* < .001), discrepancies per patient (0.86 vs 1.28, *P* < .001), and discrepancies related to missing dose or frequency (13.4% vs 38.8%, p<0.001) were all significantly lower in the pharmacist intervention group. These results highlight the beneficial effects of pharmacist presence on multidisciplinary rounds.^12^ Even more revealing, a study by Alex et al sought to identify whether a team-based approach with pharmacist intervention would influence rates of medication errors. This prospective, nonrandomized clinical trial assessed the rate of discharge-related medication errors in patients receiving pharmacist intervention (n = 145) compared to controls (n = 134). The percentage of patients without a medication error within 72 hours of discharge was significantly improved in the pharmacist intervention group (93.8% vs 40.2%, *P* < .0001), again highlighting the benefit of pharmacist involvement in a multidisciplinary care setting.^13^

The documented benefit of pharmacist involvement on multidisciplinary patient rounds necessitated their incorporation into the DCU Center’s clinical workflow.^10,11^ As part of our daily workflow, pharmacists reviewed all of the following for each patient: anticoagulant and antibiotic therapy, warfarin dosing, renal dose adjustments, and opportunities for intravenous to oral conversion for applicable medications. Pharmacists were also present on daily multidisciplinary patient rounds and contributed to discharge-based medication reconciliation discussions. Between December 2020 and March 2021, 642 patients were cared for with pharmacists implementing 668 distinct interventions, which were tracked using I-vent documentation through the Epic electronic medical record. Interventions were generally self-reported at the discretion of the pharmacist based on the individual’s clinical judgment. However, the following medication orders required intervention documentation per institutional policy: vancomycin, warfarin, direct oral anticoagulants, clozapine, and restricted antimicrobial agents. The top 3 intervention types included antimicrobial stewardship, order clarification, and daily monitoring assessment (Table 1). While objective outcomes data regarding medication errors and discrepancies are not available, the establishment of pharmacist responsibilities mimicking those in the traditional hospital setting allowed for pharmacists to implement a large number of interventions. This presumably led to optimization of medication regimens both during admission and at discharge.

**Table 1.** Pharmacist Interventions at the DCU Field Hospital.^a^

Intervention type	Number (%) interventions (n = 668)
Antimicrobial stewardship	228 (34.2)
Restricted antimicrobials	225 (33.7)
Other	2 (0.3)
Empiric therapy recommendations	1 (0.1)
Order clarification	178 (26.7)
Non-formulary	57 (8.5)
Missing laboratory data	43 (7)
Other	37 (5.5)
Patient home medication	15 (2.2)
Missing height/weight/patient information	13 (1.9)
Duplicate therapy	7 (1)
Inappropriate therapy	4 (0.6)
Missing allergy information	2 (0.3)
Daily monitoring	100 (15)
Interventions	82 (12.3)
Warfarin	8 (1.2)
Kinetics	4 (0.6)
IV to PO conversion	3 (0.4)
Creatinine clearance	2 (0.3)
Clozapine	1 (0.1)
Therapeutic drug monitoring	76 (11.4)
Enoxaparin	29 (4.3)
Warfarin	14 (2.1)
Direct oral anticoagulant	13 (1.9)
Other	9 (1.3)
Vancomycin	7 (1)
Lab monitoring	3 (0.4)
Clozapine	1 (0.1)
Dose/frequency change	35 (5.2)
Renal	19 (2.8)
Optimize therapy	8 (1.2)
Other	8 (1.2)
Education	14 (2.1)
Nurse education	7 (1)
Diabetic teaching	6 (0.9)
Other	1 (0.1)
Drug/product change	13 (1.9)
Other	6 (0.9)
Formulary interchange	3 (0.4)
Cost effectiveness	2 (0.3)
Drug shortage	1 (0.1)
Route of administration	1 (0.1)
Medication list education/review	7 (1)
Add/discontinue medication	6 (0.9)
Other	2 (0.3)
Optimize therapy	2 (0.3)
Inappropriate therapy	1 (0.1)
Duplicate therapy	1 (0.1)
Formulary interchange	3 (0.4)
Cost information	2 (0.3)
Medication reconciliation	2 (0.3)
Clinical practice guideline adherence	1 (0.1)
IV to PO	1 (0.1)
Pharmacy consult	1 (0.1)
Policy infringement	1 (0.1)

a December 6th, 2020 to February 27th, 2021.

## Implementing Remdesivir Therapy in a Field Hospital

On May 1, 2020, the FDA approved remdesivir under an Emergency Use Authorization for COVID-19 treatment and on October 22, 2020, for adult and pediatric patients 12 years of age and older and weighing at least 40 kg with COVID-19 requiring hospitalization. Remdesivir, a novel nucleotide analog, is the first drug found to have a beneficial effect on hospitalized patients with COVID-19. It works by preventing viral replication via inhibiting RNA dependent, RNA polymerase with in vitro and in vivo inhibitory activity against SARS-CoV-2 in rhesus macaque monkeys.^14^ The clinical benefit of remdesivir is mixed, resulting in a lack of consensus among society and organizational guidelines. Remdesivir has not shown a mortality benefit for patients with severe COVID-19. One subgroup analysis from a randomized, placebo-controlled trial did find a mortality benefit with remdesivir for patients who required non-high-flow oxygen, however after methodological assessment there was no statistical significance found, reducing the reliability of results.^15-18^ Remdesivir does reduce time to clinical improvement when given early in the course of illness and/or in patients with less severe disease, defined as requiring non-invasive oxygen supplementation.^14-18^

At the field hospital, remdesivir required approval from the infectious disease (ID) service who reviewed each COVID-19 patient individually. Remdesivir is not recommended in patients with an estimated glomerular filtration rate (eGFR) <30 mL/min per 1.73 m^2^ as it is prepared in a cyclodextrin vehicle that accumulates in renal impairment and may be toxic.^19^ Additionally, remdesivir is not recommended if liver enzymes; alanine aminotransferase elevations >5 times the upper limit of normal.^19^ Due to decreased efficacy, remdesivir was not used if patients were concomitantly on hydroxychloroquine or chloroquine. Once ID approved remdesivir for a patient, the pharmacist on duty ensured the antiviral was ordered with a 30-mL bolus of normal saline following each administered dose. The adult remdesivir dose is 200 mg intravenously on day 1 followed by 100 mg daily for 5 days total. If a patient was otherwise ready for discharge prior to completion of the course, remdesivir was discontinued.

The beyond use stability of remdesivir is 48 hours refrigerated which imposes challenges in a satellite pharmacy without a sterile compounding cleanroom.^20^ Due to this limitation, the pharmacist staffing at the field hospital had to coordinate with inpatient pharmacy services at the local hospital campus to compound remdesivir doses for the number of patients receiving treatment. The process of determining the number to remdesivir doses required intensive planning as to maintain enough doses to treat every patient for 24 hours while minimizing waste. The pharmacist was also responsible for the daily monitoring of renal function and hepatic enzymes.

To date, the field hospital has treated 304 patients with remdesivir and batched 866 bags since opening. As therapeutic agents continue to evolve for COVID-19, including dexamethasone and tocilizumab, we believe remdesivir will continue to be a specific treatment for this disease.^21^

## The Dual Pandemic: COVID-19 and Diabetes Raising Challenges in a Field Hospital Setting

The COVID-19 pandemic has presented numerous challenges for clinicians, including management of concomitant disease states. Over the past year, many studies have suggested a bidirectional link between diabetes and COVID-19.^22,23^ On one hand, patients with preexisting diabetes are at an increased risk of viral infection, including severe COVID-19 infection, due to alterations in innate immunity.^24,25^ Poor glycemic control can also increase infection risk in diabetic patients.^26^ On the other hand, patients with COVID-19 and no known history of diabetes are presenting with new onset diabetes and severe metabolic complications, including diabetic ketoacidosis.^25^ Possible mechanisms to explain new-onset diabetes in COVID-19 patients include binding of the SARS-CoV-2 virus to angiotensin-converting enzyme 2 (ACE2) receptors, which are expressed on pancreatic beta cells. Therefore, the SARS-CoV-2 virus may enter islets using ACE2 as its receptor and cause alterations in glucose metabolism that cause acute diabetes.^25,27^

Proper management of both new-onset and existing diabetes in COVID-19 patients is imperative given the higher rate of mortality observed in this patient population.^28^ Specifically, patients with newly diagnosed diabetes have a higher risk of all-cause mortality compared to COVID-19 patients with known diabetes.^29^ However, Zhu et al^30^ showed that among patients with COVID-19 and diabetes, well-controlled blood glucose (glycemic variability within 70-180 mg/dL) was associated with markedly lower mortality compared to individuals with poorly controlled blood glucose (upper limit of glycemic variability >180 mg/dL). Guidelines for the management of diabetes in hospitalized patients with COVID-19 recommend continuous and reliable glycemic control.^22^ Insulin is the preferred glycemic control agent for hospitalized patients, and reports have shown patients with severe COVID-19 have exceptionally high insulin requirements.^22,23^ Patients should be managed with individualized insulin therapy to target a plasma glucose concentration between 72 and 180 mg/dL. Regular monitoring of blood glucose every 2 to 4 hours should be encouraged. Other recommendations include holding metformin and sodium-glucose-co-transporter 2 inhibitors secondary to the high risk of renal dysfunction and dehydration in acute illness. Laboratory monitoring should include pH, blood ketones, and electrolytes, specifically potassium in the setting of insulin treatment.^22^

The trends of increased mortality in COVID-19 patients with diabetes prompted pharmacists at the field hospital to review all patients with diabetes to ensure they were being managed according to current guidelines.^22^ Although other comorbid disease states (ie, hypertension) are also associated with increased mortality in COVID-19,^31^ we focused our interventions on diabetic patients because delays in discharge due to diabetes teaching were observed prompting provider inquiry regarding pharmacy involvement in this process. Over the course of 3 months, we treated 207 patients with diabetes and dispensed 19 glucometers. As part of our daily chart review, clinical pharmacists reviewed diabetic regimens and recommended changes to insulin regimens as appropriate to help patients achieve their glycemic targets. We also determined whether patients had preexisting or new-onset diabetes to help facilitate glucometer and insulin teaching for patients new to these therapies. Although teaching was primarily performed by nursing staff, pharmacists obtained diabetes prescriptions from the outpatient pharmacy and coordinated teaching sessions with the interprofessional team. While the full impact of pharmacist diabetes interventions at the field hospital is not fully known, we were able to help optimize the glycemic regimens of patients followed by the pharmacy service.

## Conclusion

The pharmacist role at the field hospital setting expanded more than expected with the second surge in the COVID 19 pandemic. From coordination with outpatient pharmacy services ensuring patients receive their own medications and diabetes testing supplies, to coordination with inpatient pharmacy services to ensure patients receive medications including remdesivir treatment. Having a satellite style pharmacy that had to be capable of providing services equivalent to that of an acute care level hospital pharmacy was challenging, but also came with opportunity to make improvements to patient care.
